# A reverse-engineering approach to building our understanding of nervous systems

**DOI:** 10.1186/1471-2202-15-S1-P28

**Published:** 2014-07-21

**Authors:** Herve Thevenon

**Affiliations:** 1 Imezio Ltd, Wellington, New Zealand

## 

Neuroethological studies’ pace of progress is currently hindered by three major factors: (a) there isn’t any species for which we have a nearly perfect knowledge of both the connectome and the neuronal characteristics; (b) while we can simulate realistically some fractions of specific species nervous system, the simulations lack the capability to interact with the species’ natural environment or a sufficiently detailed simulation of that specific environment; (c) we lack the tools to disconnect and reconnect neurons at will during in vivo experiments to improve and confirm our simulations.

The history of science is full of simple theories and models that paved the way for better ones. Our view is that the enormous complexity involved in creating realistic models of living nervous systems and their natural environment should not stop us trying to create simple simulations as a whole. New theories shall naturally flow from them and any refutation will improve the current state of knowledge. We postulate that evolutionary algorithms have the capability to fill in the blanks where we lack hard data in order to create simulated nervous systems that exhibit the behavioral characteristics of their real life models.

In order to test the feasibility of our concept, we have built a dynamic environment that can instantiate avatars of nervous systems modeled upon a set of characteristics observed in the living [1] and evolved using an evolutionary algorithm to improve their life expectancy by mutation. The nervous systems are independent from one another, and interact with the environment via a dedicated set of 7 binary sensors (corresponding to 5 different senses) and 3 actuators. While the interactions are essentially asynchronous, the environment contains a mechanism to ensure that all nervous systems can communicate with it at the same rate. The characteristics included in our simulation of nervous systems include: firing patterns of variable duration and intensity allowing for action potentials and plateaux [2], refractory periods, spatial location of the neurons, inhibitory and excitatory connections, and self firing neurons [3]. Using *C. elegans* as a model of reference, we conjectured that 30 of these neurons would be enough to observe the emergence of non random behaviors for this amount of sensors and actuators. On this basis, the number and the range of the variables used to define the nervous systems create a search space of order 10^43^. This order of magnitude is the main reason for using evolutionary techniques to evolve nervous systems, as they allow to finding solutions without prior knowledge.

In our experiment, the individual that survived the longest used 27 neurons, 2 out of the 3 possible actuators, and exhibited a variety of reflexes. The whole palette of firing patterns is used, but out of 160 connections only 4 are inhibitory. Despite its simplicity, this simulation confirmed the feasibility of the concept, and allowed us to build metrics around the computational requirements for more complex simulations that will feature analog sensory inputs in order to try and observe other innate behaviors: taxes, kineses, and habituation [4].
